# Kostenfreie Onlinekurse nachhaltig mit personalisiertem Marketing finanzieren – Ein Vorschlag zur synergetischen Kombination zweier datengetriebener Geschäftsmodelle

**DOI:** 10.1365/s40702-021-00720-4

**Published:** 2021-04-09

**Authors:** Sylvio Rüdian, Gergana Vladova

**Affiliations:** 1 Weizenbaum-Institut, Humboldt-Universität zu Berlin, Unter den Linden 6, 10117 Berlin, Deutschland; 2 Weizenbaum-Institut, Universität Potsdam, Am Neuen Palais 10, 14469 Potsdam, Deutschland

**Keywords:** Onlinekurse, Big Data, Geschäftsmodell, Werbung, Marketing, Canvas, Online courses, Big data, Business model, Advertisement, Marketing, Canvas

## Abstract

Selbstbestimmtes Lernen mit Onlinekursen findet zunehmend mehr Akzeptanz in unserer Gesellschaft. Lernende können mithilfe von Onlinekursen selbst festlegen, was sie wann lernen und Kurse können durch vielfältige Adaptionen an den Lernfortschritt der Nutzer angepasst und individualisiert werden. Auf der einen Seite ist eine große Zielgruppe für diese Lernangebote vorhanden. Auf der anderen Seite sind die Erstellung von Onlinekursen, ihre Bereitstellung, Wartung und Betreuung kostenintensiv, wodurch hochwertige Angebote häufig kostenpflichtig angeboten werden müssen, um als Anbieter zumindest kostenneutral agieren zu können. In diesem Beitrag erörtern und diskutieren wir ein offenes, nachhaltiges datengetriebenes zweiseitiges Geschäftsmodell zur Verwertung geprüfter Onlinekurse und deren kostenfreie Bereitstellung für jeden Lernenden. Kern des Geschäftsmodells ist die Nutzung der dabei entstehenden Verhaltensdaten, die daraus mögliche Ableitung von Persönlichkeitsmerkmalen und Interessen und deren Nutzung im kommerziellen Kontext. Dies ist eine bei der Websuche bereits weitläufig akzeptierte Methode, welche nun auf den Lernkontext übertragen wird. Welche Möglichkeiten, Herausforderungen, aber auch Barrieren überwunden werden müssen, damit das Geschäftsmodell nachhaltig und ethisch vertretbar funktioniert, werden zwei unabhängige, jedoch synergetisch verbundene Geschäftsmodelle vorgestellt und diskutiert. Zusätzlich wurde die Akzeptanz und Erwartung der Zielgruppe für das vorgestellte Geschäftsmodell untersucht, um notwendige Kernressourcen für die Praxis abzuleiten. Die Ergebnisse der Untersuchung zeigen, dass das Geschäftsmodell von den Nutzer*innen grundlegend akzeptiert wird. 10 % der Befragten würden es bevorzugen, mit virtuellen Assistenten – anstelle mit Tutor*innen zu lernen. Zudem ist der Großteil der Nutzer*innen sich nicht darüber bewusst, dass Persönlichkeitsmerkmale anhand des Nutzerverhaltens abgeleitet werden können.

## Abstract (en)

Self-determined learning is increasingly accepted in our society. Online courses offer scheduling flexibility and pacing options, so that the learners can complete their assignments at convenient times. Moreover, online courses can be adapted and individualized to match the learning progress of the users through various adaptations. There is a large target group that aims to develop certain skills with online courses. But their creation, implementation, maintenance, and support are cost-intensive, which means that high-quality tutored courses often have to be offered fee-based to operate at least cost-neutrally without gaining some profit. In this paper, we demonstrate and discuss an open, sustainable, data-driven two-sided business model for using verified online courses and making them available to every learner free of charge. The core of the business model is the use of behavioral data for deriving personality traits and interests, and their use in a commercial context. This method is already widely accepted in web search, which is now implemented in the learning context. The opportunities, challenges, but also barriers that need to be overcome for the business model to be applicable and ethical at the same time are discussed while two independent but synergistically connected business models are presented. Besides, we examined the acceptance and expectation of the target group for the presented business model to understand the necessary core assets for practice. The results of the study indicate that the business model is fundamentally accepted. 10% of the respondents prefer to learn with virtual assistants instead of tutors, and the majority of users is not aware that personality traits can be derived from user behavior.

## Einführung

Onlinekurse haben seit den letzten Jahrzehnten eine zunehmend höhere Beliebtheit erlangt. Sie ermöglichen ein selbstbestimmtes, zeitlich und örtlich unabhängiges Lernen in einer virtuellen Umgebung. In Zeiten von COVID-19 wurde deutlich, wie wichtig Online-Lernangebote sind und viele sind bereit, diese alternative Form zum Präsenzlernen zu akzeptieren und zu nutzen, sowohl im universitären Kontext als auch zum Zweck der Weiterbildung durch Selbstlernangebote. Als Antwort auf die steigende Nutzernachfrage wachsen parallel sowohl die Märkte in diesem Bereich als auch die Zielgruppe. Dabei entstehen Online-Lernangebote häufig im kommerziellen Kontext mit einer direkten Absicht zur Gewinnerzielung. Die Geschäftsmodelle (GM) sind vielfältig und reichen von Freemium- und Abo-Modellen über tutorierte Onlinekurse bis hin zu individuellen Kursen im Kontext von Weiterbildungen. Eine Nutzung der beim Lernen entstehenden Daten der Lernenden zum Zweck des Marketings erfolgt bislang nicht.

In diesem Beitrag stellen wir ein zweiseitiges GM vor, welches klassische Online-Lernangebote mit einem datengetriebenen GM zur Ausspielung nutzerspezifischer Werbung kombiniert. Die grundlegende Motivation dieses GM ist vielschichtig und auf einen nicht kommerziellen Kontext ausgerichtet, in welchem Onlinekurse entstehen. Zum einen werden im Rahmen von Forschungsprojekten häufig Finanzierungen für einen konkreten Zeitrahmen zur Verfügung gestellt, welche die Erstellung von Onlinekursen als Förderziel haben. Nach Ablauf der Förderperiode stellt sich die Frage der künftigen Nutzung, die von der anschließenden Finanzierung durch geeignete Partner abhängig ist. Findet sich kein Partner, werden solche Projekte eingestellt. Im anderen Fall erfolgt eine kommerzielle Ausrichtung der Projekte, sodass das primäre Vorhaben, beispielsweise Onlinekurse den Endanwendern kostenfrei zur Verfügung zu stellen, scheitert. Dabei entstehen an Universitäten und Forschungseinrichtungen durch geförderte Projekte viele sehr hochwertige Beiträge, deren Nutzung durch die Öffentlichkeit möglich sein sollte (BMBF 2020). Einen zweiten nicht kommerziellen Kontext bilden Onlinekurse, welche im Rahmen von Seminaren durch Studierende erstellt wurden mit dem Ziel, ihre Kompetenzen zur kritischen Begutachtung, konzeptuellen und technischen Umsetzung bereits im Studium zu entwickeln (Vladova und Rüdian 2020). Gerade im Rahmen des Studiums entsteht seitens der Studierenden viel Output – beispielsweise in Form von Seminararbeiten – der jedoch nicht veröffentlicht wird. Ein selbst erstellter Onlinekurs kann als Referenz genutzt werden, wenn dieser auch nach dem Seminar Dritten zur Verfügung steht. Demnach diskutieren wir in diesem Beitrag zwei Quellen, aus denen hochwertige Onlinekurse ohne kommerziellen Hintergrund entstehen: 1) aus geförderten Drittmittelprojekten und 2) durch Studierende; und entwickeln hierzu ein zweiseitiges GM auf der Grundlage des Business Model Canvas (Osterwalder und Pigneur 2010), kurz: BMC. Das erste GM umfasst demnach die Erstellung und Verwertung von Onlinekursen, die in hoher Anzahl mit hoher Qualität bereits jetzt fortlaufend erstellt werden. Parallel dazu erstellen auch Dritte immer wieder Lerninhalte. So entwickeln Lehrende Lernvideos für ihre Schüler, aber auch weitere Lernangebote können skalierbar in Onlineformate transformiert werden. Auch wenn diese und weitere Quellen bekannt sind und gut funktionieren, besteht nach wie vor das Problem der angemessenen künftigen Bereitstellung und Verwertung. Vor dem Hintergrund dieser Problematik wird in diesem Paper das erste GM entwickelt – *Das Geschäftsmodell für die Verwertung und Bereitstellung von Onlinekursen *als Data-User-GM (Wiener et al. 2020), welches im Folgenden als GM⋅: gekennzeichnet wird.

Haben Lernende Zugriff auf die bereits entwickelten Onlinekurse und nutzen sie die Angebote zum Lernen, entstehen hierbei wie in jeder Webanwendung Daten. Jeder Klick wird aufgezeichnet und auch jede interaktive Anwendung (z. B. ein Quiz) erzeugt Daten darüber, wie gut jeder Lernende Aufgaben oder Fragen gelöst hat. In einem Forschungsbeitrag (Rüdian et al. 2019) stellten die Autoren fest, dass die Kombination der Daten in einem Onlinekurs, bezeichnet als Klickstromdaten genutzt werden können, um Persönlichkeitseigenschaften vorherzusagen. Hierzu wurde ein klassisches Neuronales Netz trainiert und mit den Konstrukten aus zwei Fragebögen kombiniert, Big‑5 (Barrick und Mount 1991) und CVSCALE (Yoo et al. 2011), um diese vorherzusagen. Dies funktioniert mit einer hohen Genauigkeit, sodass diese Technik praktisch einsetzbar ist. Die Idee der Autoren besteht darin, diese Informationen zur individuellen Adaption von Onlinekursen zu nutzen. Einem Lernenden, der beispielsweise eine hohe Persönlichkeitsausprägung der „Individualism“-Eigenschaft hat, muss keine Aufgabe vorgeschlagen werden, die es in einer Gruppe zu lösen gilt. Andere haben als Persönlichkeitsmerkmal eine erhöhte Unsicherheit. Die Kenntnis hierüber kann genutzt werden, um die Lernenden mit Hilfestellungen in Onlinekursen zu unterstützten. Diese Adaptionen können helfen, sowohl die Motivation als auch den Lernerfolg der Lernenden zu verbessern.

Die hierbei entstehenden Nutzerprofile, welche die Persönlichkeiten abbilden können, sind aus der Marketingperspektive höchst bedeutsam. Die Geschäftsmodelle großer Tech-Konzerne wie Google zeigen wie wichtig Nutzerprofile sind, um Kaufintentionen abzuleiten, die für nutzerspezifische Werbung genutzt werden können (Krys und Wiedemann 2011). Das Angebot, eine Suche für Webseiten bereitzustellen, kennzeichnet dort das sichtbare GM; doch die Nutzung der Verhaltensdaten zur Profilbildung und Anwendung im Marketing ist das allgegenwärtige kostentragende GM und wird weitläufig akzeptiert (Cabinakova et al. 2016). In Anlehnung daran schlagen wir die Nutzung der aus Onlinekursen abgeleiteten Nutzerprofile in einem GM vor, um diese zum Zweck des Marketings zur *Ausspielung nutzerspezifischer Werbung zu verwenden *(GM$). Damit können die Bereitstellung und Instandhaltung der Infrastruktur und die Betreuung der Onlinekurse aus dem GM⋅: finanziert werden. Wir nutzen die Typologie von Wiener et al. (2020) und unterscheiden zwischen „Data User“ (welcher die Onlinekurse und gewonnenen Daten für interne Zwecke nutzt) und dem „Data Supplier“ (welcher die gewonnenen Daten weiter vermarkter und monetarisiert) als zwei Grundtypen von GM. Das GM$ ist nach der Typologie von Wiener et al. (2020) ein Data-Supplier-GM, welches die entstehenden Klickstromdaten mit abgeleiteten Nutzerprofilen aus den Onlinekursen des ersten GM⋅: verwendet. Es handelt sich nicht um ein weiteres Data-User-GM, da Nutzerprofile nicht nur auf der eigenen Plattform genutzt werden sollen, sondern auch an Werbenetzwerke zum Zweck der Monetarisierung übertragen werden. Im Rahmen dieses Beitrags werden zuerst die theoretischen Grundlagen erläutert und nachfolgend umfassende Perspektiven des zweiseitigen Geschäftsmodells (GM⋅: und GM$) präsentiert und dessen Umsetzung, Machbarkeit und Akzeptanz diskutiert.

## Nutzereigenschaften als Grundlage für personalisierte Online-Werbung

In der Literatur werden verschiedene Modelle verwendet, um Nutzereigenschaften und Normen auf der persönlichen Ebene in Onlinekursen zu definieren, von denen wir uns auf zwei beschränken. Auf der einen Seite unterscheidet Hofstede Personengruppen anhand von 6 kulturellen Merkmalen: Machtdistanz, Individualismus, Unsicherheitsvermeidung, Langzeitorientierung, Maskulinität und Genuss (Hofstede 2017). Diese Normen und Präferenzen können auf der Persönlichkeitsebene erhoben werden, da die Beschreibungen der definierten Konstrukte nicht ausschließlich für Gruppen gelten. Dies wurde in der CVSCALE evaluiert (Yoo et al. 2011). Auf der anderen Seite können durch die Anwendung des Big-5-Tests (Barrick und Mount 1991) Merkmale zur Persönlichkeit abgeleitet werden (Offenheit, Gewissenhaftigkeit, Extraversion, Verträglichkeit und Neurotizismus). Persönlichkeitsmerkmale beschreiben, anders als abgeleitete Normen, Gedanken und Verhaltensmuster (Mooij 2015). Beide Modelle sind mit einer hohen Genauigkeit anhand des Nutzerverhaltens innerhalb von Onlinekursen vorhersagbar (Rüdian et al. 2019) und beide können im Lernkontext zum Zweck der Personalisierung genutzt werden.

Caliskan (2019) untersuchte, ob im Beziehungsmarketing (Relationship Marketing) zum Aufbau einer erfolgreichen Beziehung zwischen Konsument*innen und Marken die Persönlichkeitsmerkmale aus dem Big-5-Test zur Auswahl einer optimalen Strategie verwendet werden können. Die Ergebnisse haben gezeigt, dass die Big-5-Persönlichkeitseigenschaften allesamt genutzt werden können, um die für Konsument*innen optimalen Strategien zu wählen. Es gibt zahlreiche Studien, welche Marketingstrategien bezogen auf Persönlichkeitsmerkmale untersucht haben. Hirschman (1980) zeigte beispielsweise, dass die hohe Offenheit für neue Erfahrungen dazu führt, dass jene Personengruppe eher bereit ist, neue Produkte anzunehmen. Neurotizismus mit einer geringen emotionalen Stabilität hat zur Folge, dass jene Personengruppen weniger Vertrauen haben und kritisch gegenüber Neuem sind (Freitag und Bauer 2016). Personen mit hoher Gewissenhaftigkeit sind sehr organisiert, planen ihre Ausgaben und benötigen detaillierte Informationen, um eine Entscheidung treffen zu können (Mondak 2010). Die Big-5-Persönlichkeitsmerkmale haben gemäß Guido (2006) eine hohe Korrelation zu hedonischen und utilitaristischen Kaufmetriken (Ahtola 1985). Deren Kenntnis ist im Marketing äußerst nützlich, um die Effizienz zu erhöhen und nutzerspezifische Marketingstrategien anzuwenden. Es ist daher in vielen Szenarien nicht optimal, lediglich anhand demographischer Daten Werbemaßnahmen zu personalisieren. Die Nutzung von Merkmalen auf der Persönlichkeitsebene ermöglicht neue Perspektiven für das Marketing.

Während die kulturelle Skala von Hofstede (2011) lediglich den Durchschnitt von Personengruppen erfasst, welcher für den Einsatz im Marketing immer wieder kritisiert wird (Venaik und Brewer 2013), liefert die CVSCALE Normen auf der Persönlichkeitsebene, welche auch in Korrelation zu den Big‑5 Merkmalen stehen (Chien et al. 2016). Gemäß Jagodzinsky (2004) fokussieren wir damit den Bereich der Mikro-Ebene. Wird lediglich das Herkunftsland der Lernenden als Basis zur Ableitung von Normen und Präferenzen gemäß Hofstede (2011) verwendet und die dort zum Vergleich von Kulturen genutzten Charakteristika der individuellen Ebene gleichgesetzt, führt das aufgrund der hohen Streuung innerhalb der kulturellen Gruppen zu starken Abweichungen (Rüdian und Gundlach 2021). Onlinekurse nur auf Länderebene zu optimieren kann viele Personengruppen systematisch ausschließen, da die kulturelle Ebene nicht mit der individuellen Ebene gleichgesetzt werden kann (Fischer und Poortinga 2012). Daher sollte der Transfer von länderspezifischen kulturellen Eigenschaften auf das Individuum vermieden werden (Taras et al. 2010), da sonst fehlerhafte Schlussfolgerungen getroffen werden können. Eine Skala für Konstrukte auf der individuellen Ebene, welche die CVSCALE liefert, ist daher notwendig. Besonders bedeutsam ist die Verbindung zwischen persönlichen Wertevorstellungen und Verhaltensweisen, durch welche sich in einem gewissen Grad das Verbraucherverhalten vorhersagen lässt (Mooij 2015). Für nutzerspezifisches Marketing ist die Kenntnis darüber ein Anhaltspunkt, Nutzende in Segmente zu überführen, um möglichst effiziente Marketingstrategien zu verfolgen.

Bezogen auf die Aktivität innerhalb von Onlinekursen kann zudem der Wissensstand in einem Modell abgebildet werden, um den Lernpfad zu personalisieren (Brusilovsky et al. 2005). Dies ist ein wichtiger Kernaspekt adaptiver Onlinekurse, um zeiteffizient zu lernen und durch Automatismen bereits bekanntes Wissen nicht erneut grundlegend lernen zu müssen, was bei einem fest definierten Lernpfad der Fall ist. Zudem kann ein solches Wissens-Modell genutzt werden, um Empfehlungsalgorithmen anzuwenden, welche einen effizienten Zugang zu potenziell interessanten und dem Lernenden (noch) unbekannten Onlinekursen ermöglichen (Drachsler et al. 2008). Je nach Motivation zur Teilnahme an Onlinekursen kann das Wissen von Lernenden aus der Marketingperspektive genutzt werden, um Interessen abzuleiten. Ist das Lernangebot inhaltlich vielfältig und breit gestreut, lassen sich situative Merkmale ableiten, um zum Beispiel berufliche Angebote zu bewerben. Decken Kurse abseits der akademischen Welt Themen ab, welche Hobbies der Lernenden behandeln, lassen sich hier ebenfalls Interessen ableiten, um sie einem Profil hinzuzufügen.

## Synergetische Kombination zweier datengetriebener Geschäftsmodelle

Im Folgenden verwenden wir das „Business Model Canvas“ (Osterwalder und Pigneur 2010), um die zwei oben eingeführten GMs zu entwickeln. Das BMC ist „eine gemeinsame Sprache zur Beschreibung, Visualisierung, Bewertung und Veränderung von Geschäftsmodellen“ (Osterwalder und Pigneur 2010). Es wurde mit dem Ziel entwickelt, Organisationen und Manager dabei zu unterstützen, ein Modell über die relevanten Aktivitäten zu entwerfen, um es in der gesamten Organisation zu kommunizieren. Das Modell berücksichtigt vier grundlegende Geschäftsbereiche sowie relevante Einflussgrößen (Osterwalder und Pigneur 2010). Zusammengefasst unterstützt das Modell die Analyse und Darstellung, wobei andere Instrumente zur Analyse und Quantifizierung die Ergebnisse erweitern können. Das BMC wurde in diversen Praxiskontexten angewandt, unter anderem im Kontext der Digitalisierung (Heberle et al. 2017) sowie im universitären Kontext (Rytkönen und Nenonen 2014).

Unser zweiseitiges GM besteht aus dem Data-User-GM (bezeichnet als GM⋅:) und Supplier-GM (bezeichnet als GM$). Die GMs unterscheiden sich grundlegend in ihrer Ausrichtung, Zielgruppe und Aktivitäten, sind jedoch als Symbiose notwendig, da GM⋅: in der vorgestellten Form nicht kostendeckend agieren kann und GM$ die essenzielle Datengrundlage zur Profilbildung für nutzerspezifische Werbung fehlt. Daher ist eine Kombination beider Modelle in einem Gesamtkonzept notwendig, die mit Vorteilen bei ihrer Nutzung einhergeht. Solche Geschäftsmodell-Synergien adressieren die positiven Auswirkungen als Folge davon, dass zwei in sich eigenständige und getrennte Geschäftsmodelle miteinander verbunden werden, wie beispielweise bei der Verbindung von digitalen und nicht-digitalen GM in Unternehmen (vgl. Toutaoui et al. 2021).

**Abb. 1:**
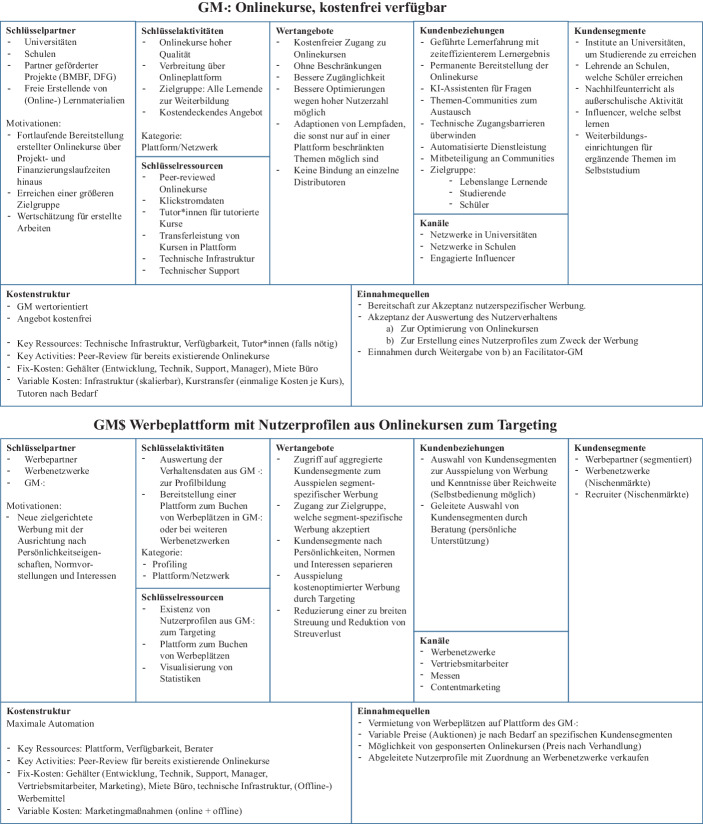
Business Model Canvas der GMs *Data User* als GM⋅: und *Data Supplier* als GM$

Grundlage für die Konzeption des zweiseitigen GMs ist das Ergebnis von Experteninterviews mit je drei Vertretern der Lehrenden, Lernenden, Wirtschaftsinformatikern und Betriebswirtschaftlern, welche jeweils 45 min dauerten. Die Experten lieferten vor dem Hintergrund ihres konkreten Fachwissens direkte Hinweise, die notwendig waren, um den interdisziplinären Herausforderungen der GM-Entwicklung gerecht zu werden. Sowohl die Anwender- und Nutzerperspektive als auch die Wirtschaftlichkeit und die konkrete technische Umsetzung wurden in der Konzeptionsphase adressiert. Die entstandenen BMC sind in Abb. 1 separat dargestellt.

Die Basis für GM⋅: ist die Existenz hochwertiger Onlinekurse, die durch Projekte entstehen, welche öffentlich finanziert wurden, jedoch in ihrem Dasein häufig zeitlich limitiert sind. Parallel entstehen an Universitäten und Hochschulen tausende Onlinekurse, deren Entwicklung durch die COVID-19 Krise noch weiter vorangetrieben wurde. Auch an Schulen entstehen Onlinekurse, um Schülerinnen und Schülern das Lernen von Zuhause aus zu ermöglichen. Dank moderner Technologien wie H5P lassen sich viele Konzepte einfach technisch umsetzen. Seitens der Erstellenden besteht ein hohes Bedürfnis, dass die erstellen Kurse auch nach Ablauf der Projektlaufzeiten weiterhin ein breites Zielpublikum erreichen und keine fortlaufende administrative Arbeit entsteht. Diese Kurse werden in eine moderne Onlinekursplattform übertragen und gebündelt. Während für gängige Onlinekursanbieter die Erstellung einer der größten Kostenfaktoren ist, können wir auf existierendem Material aufbauen. Kurse können anhand von Leitfäden in einem Peer-Review-Verfahren evaluiert werden – sodass für den Fall, dass identische Themen mehrfach abgedeckt werden, die bestmögliche Version identifiziert und verwendet wird. Die wichtigsten Ressourcen sind die bestehenden Onlinekurse und eine Plattform mit Infrastruktur, auf welche die Kurse übertragen werden können. Aber auch Tutor*innen sind ein wichtiger Faktor, welche die Lernenden begleiten und nutzerspezifisches Feedback geben können, welches noch nicht vollständig von KI-Methoden abgedeckt werden kann (VanLehn 2006). Dies ist vor allem für Lernangebote notwendig, welche nicht automatisiert ausgewertet werden können. Durch das Angebot an hochwertigen kostenfreien Onlinekursen „free of charge“ (Schüritz et al. 2017) für die Lernenden wird der Zugang für die Zielgruppe erleichtert, was einen hohen Wettbewerbsvorteil verspricht. Die Schwelle zur Teilnahme ist demnach niedrig angesetzt, wodurch ein schnelleres Wachstum erzielt werden kann als durch kostenpflichtige Lernangebote. Grundlegend ist das GM⋅: wertorientiert, erzeugt jedoch Kosten, deren größter Anteil sowohl aus der Bereitstellung der Infrastruktur bestehen als auch aus dem Angebot der Tutorierung, deren Notwendigkeit in einer quantitativen Befragung überwiegend bestätigt wurde (siehe Kap. 4).

Der im GM⋅: entstehende Wert ist die Sammlung der Nutzerdaten. Das GM⋅: agiert demnach als Datenlieferant. Jene Daten sind die Grundlage für das GM$. Das kostentragende GM$ ist darauf ausgerichtet, das GM⋅: vollumfassend zu finanzieren. Hierzu werden aus den Nutzerdaten weitere personenbezogene Metadaten abgeleitet, z. B. Persönlichkeitseigenschaften und Interessen. Die dabei abgeleiteten Nutzerprofile sind die Basis, um nutzerspezifische Werbung – innerhalb der Lernplattform und in dritten Werbenetzwerken – zu ermöglichen. Werbenden können verschiedene Kundengruppen, aufbereitet nach Segmenten, angeboten werden, um nutzerspezifisch anhand einer Vielzahl von Merkmalen Werbung ausspielen zu können. Diese Methode reduziert gezielt den Streuverlust und verringert die Kosten. Gemäß Schüritz et al. (2017) handelt es sich hierbei um „Ensure-Ads“ kombiniert mit dem Ansatz „Pay-with-data“. Parallel können abgeleitete Nutzerprofile an Werbenetzwerke übertragen werden, welche die Lernenden der Plattform auch auf dritten Webseiten identifizieren und dort ebenfalls nutzerspezifisch werben können. Da die Werbeplätze innerhalb der eigenen Lernplattform begrenzt sind, können die Preise je nach Wettbewerb variabel gehalten und in Echtzeit über Auktionen gehandelt werden. Dies sorgt für eine optimale Preisgestaltung mit maximaler Gewinnorientierung für das GM$. Nebenläufig können Produktplatzierungen für bestehende Onlinekurse als weitere Einnahmequelle akquiriert werden, welche in die Onlinekurse des GM⋅: integriert werden können.

Da das GM$ sehr kostenorientiert agiert, ist es essenziell, dieses vom GM⋅: zu trennen. Ohne GM⋅: kann das GM$ jedoch nicht existieren, da sonst der Datenlieferant für das datengetriebene Geschäftsmodell fehlt. Würden Onlinekurse aus dem GM$ entwickelt oder ausgewählt, würden dabei nur jene mit maximaler Reichweitengröße identifiziert werden, da dies zur Gewinnmaximierung führt. Daher darf eine Selektion aus ethischer Sicht nicht von einer Gewinnmaximierung abhängen und dies erfordert ein separates GM, wobei das kostentragende GM$ keinen inhaltlichen Einfluss auf die Onlinekurse haben darf. Dies ist ein wichtiger Faktor, um hochwertige Onlinekurse anbieten zu können, welche nicht ausschließlich die Interessen werbender Firmen vertreten.

## Akzeptanz und Erwartungen

Um die Akzeptanz des in diesem Paper vorgestellten zweiseitigen GM zu testen, haben wir 750 Personen befragt, um die Bereitschaft zum Teilen von Daten und deren ökonomische Verwertung, die Notwendigkeit von Tutoren und das Verständnis zum Stand der Technik zu ermitteln. Die Teilnehmenden wurden mittels Prolific nach bestimmten Kriterien akquiriert: Sie sollten mindestens eine Genehmigungsrate von 95 % aufweisen und mindestens an 500 vorherigen Studien teilgenommen haben. Jeder Teilnehmende erhielt für den Aufwand von 6 min eine Entschädigung von 0,60 €. Um automatisierte Bots zu filtern, wurde die Studie mittels ReCaptcha abgesichert. Die Fragen und die deskriptive Auswertung der Ergebnisse wurden in Tab. 1 als Ergebnis zusammengefasst, woraus sich Trends ablesen lassen.

**Tab. 1:** Antworten auf Fragebogen zur Akzeptanz des zweiseitigen GM

	ID	Frage	Stimme zu (%)	Lehne ab (%)	Neutral (%)
Ökonomisch	F1	Wenn ein Onlinekurs kostenlos ist, bin ich bereit, meine Nutzerdaten im Austausch zur Verfügung zu stellen	42,3	26,8	30,9
	F2	Ein bezahlter Kurs ist besser als ein kostenloser	19,2	36,4	44,4
	F3	Um die Erstellung von Onlinekursen zu finanzieren, sind Produktplatzierungen in Kursen für mich in Ordnung	45,6	31,2	23,2
Tutoren	F4	Künstliche Intelligenzen werden Tutor*innen ersetzen	21,3	56,9	21,7
	F5	Ich bevorzuge mit virtuellen Assistenten (KI) zu lernen, anstelle mit einer/einem persönlichen Tutor*in	10,1	64,8	25,1
KI	F6	Zukünftig werden Onlinekurse durch Künstliche Intelligenzen erstellt	52,1	18,4	29,5
	F7	Aufgrund meines Verhaltens in einem Onlinekurs kennt eine KI Details über meine Persönlichkeit	28,7	45,3	26,0

Es ist bekannt, dass die Bereitstellung eines Service (beispielsweise einer Onlinesuche) mit der verbundenen Bereitstellung von Nutzerdaten und deren Verwertung zum Zweck des Marketings weitläufig akzeptiert wird (Cabinakova et al. 2016). Darauf zielt Frage 1 (F1) ab, um die generelle Akzeptanz des zweiseitigen GMs zu überprüfen. Mehr Teilnehmende akzeptieren diese Methode, nur etwa ein Viertel lehnt es ab. F2 überprüft, ob eine Erwartung besteht, dass Kurse, bei denen die Teilnahme kostenpflichtig ist, besser sind als kostenlose Varianten; ohne das „besser“ als Gütemaß zu konkretisieren. Die Antworten zeigen eine deutliche Ablehnung des Statements. Demnach wird das Angebot von kostenfrei nutzbaren Onlinekursen aus dem GM⋅: nicht grundlegend abgelehnt und die Hypothese, dass kostenfreie Onlinekurse mit einer geringeren Qualität gleichgesetzt wurden, hat sich nicht bestätigt. Die Mehrheit akzeptiert in F3 die Finanzierung zur Erstellung von Onlinekursen durch Produktplatzierungen als gängige Methode aus der TV-Unterhaltungsbranche, welche auf Onlinekurse übertragen wurde. Demzufolge kann das GM$ das kommerzielle Angebot erweitern und Onlinekurse „sponsoren“ lassen, wenn im Austausch Produkte in den Kursen platziert werden. Das Geschäftsmodell vertritt die ökonomischen Interessen eines Großteils der Teilnehmenden.

Ob tutorierte Onlinekurse angeboten werden sollten oder solche, welche vollständig automatisiert ausgewertet werden, wird durch die Fragen F4 und F5 deutlich. Es besteht generell keine Erwartung, dass Tutor*innen durch Verfahren künstlicher Intelligenzen ersetzt werden. Zudem lehnt der Großteil der Befragten einen virtuellen Assistenten im Onlinekurs ab und präferiert die Begleitung durch persönliche Tutor*innen. Demnach ist der Einsatz von Tutor*innen für den Großteil obligatorisch und sollte als Kostenfaktor in GM$ berücksichtigt werden. Die Begleitung durch Tutor*innen mindert die Skalierungsmöglichkeiten des GM$, wird jedoch von den Teilnehmenden bevorzugt. Trotzdem sind 21 % der Ansicht, Tutor*innen werden in Zukunft ersetzt und 10 % bevorzugen, mittels virtuellen Tutor*innen zu lernen. Bei der großen absoluten Menge an Lernenden als Zielgruppe ist diese (Teil)-Gruppe jene, die hoch skalierbar adressiert werden kann, da die limitierenden Ressourcen – die Tutor*innen – hier nicht bestehen.

Die Teilnehmenden sind in F6 überwiegend der Ansicht, dass Onlinekurse künftig durch Verfahren künstlicher Intelligenzen erstellt und generiert werden. Dies ermöglicht eine personalisierte Lernerfahrung. Zum Stand der Technik ist die Personalisierung auf die Makro-Ebene beschränkt, um beispielsweise identifizierte Wissenslücken zu beseitigen. Eine Personalisierung anhand der didaktischen Methodik auf der Meso-Ebene zur Vermittlung von Inhalten je nach individuellen Präferenzen ist Gegenstand aktueller Forschung. Die Optimierung der Mikro-Ebene, um gestalterische Elemente innerhalb von Kursen zu personalisieren ist bislang nicht möglich, da die Teilnehmendenzahlen zu gering sind, um statistisch valide Entscheidungen treffen zu können. F7 prüft die Erwartung der Teilnehmenden, ob anhand des Nutzerverhaltens Persönlichkeitsmerkmale abgeleitet werden können. Der Großteil ist der Ansicht, dass dies nicht möglich ist. Jedoch haben Rüdian et al. (2019) gezeigt, dass hierbei sogar eine sehr hohe Genauigkeit erzielt werden kann, sodass kein Persönlichkeitstest notwendig ist, um die Profile zu erheben.

## Diskussion & Ausblick

In diesem Beitrag wurde ein zweiseitiges GM konstruiert, welches es erlaubt, in einem nicht kommerziellen Kontext Onlinekurse primär zu verwerten, zu verbreiten und durch gezieltes Marketing eine finanzielle Grundlage für die nachhaltige Bereitstellung und Weiterentwicklung zu erschaffen. Durch eine Umfrage wurden das Verständnis und die Akzeptanz des zweiseitigen GMs ermittelt.

Die vorgestellten Geschäftsmodelle bieten diverse Vorteile: Die Bereitstellung und Bündelung hochwertiger Lernangebote auf einer Lernplattform ermöglichen bessere Anpassungsmöglichkeiten von Lernpfaden, da zum einen eine breite Grundbasis an Kursen besteht und zum anderen erwartet werden kann, dass die kostenlose Nutzung mehr Lernende ansprechen wird und dadurch mehr Nutzerdaten entstehen werden. Diese können als Trainingsdaten für die Anwendung von Verfahren der Künstlichen Intelligenz genutzt werden, und so die Basis für automatisierte Adaptionen bilden. Durch die hohe Menge entstehender Daten kann ein sehr reichhaltiges Wissensmodell der Lernenden abgeleitet werden, welches aufgrund der Vielzahl angebotener Kurse deutlich mehr Informationen enthalten kann als es bei Anbietern mit weniger Angeboten der Fall ist. Dies ermöglicht Empfehlungen von Inhalten, welche durch Nischenanbieter gar nicht abgedeckt werden. Es ist bekannt, dass nutzerspezifische Werbung weitgehend positiv wahrgenommen wird, wenn die ausgespielten Inhalte als nützlich eingestuft werden (Merisavo et al. 2007). Wird somit die Akzeptanz ausgespielter Werbung erhöht, ist dies ein positiver Faktor, sowohl für die Lernenden als auch aus der Perspektive des Marketings. Die entstehenden Daten aus dem GM⋅: bilden dafür die Grundlage.

Werden Studierende als Kursentwickelnde im Rahmen ihrer Hochschulbildung aktiv, profitieren sie durch den Erwerb von Medien- und digitalen Kompetenzen. Zeitgleich können entstandene Onlinekurse durch Studierende als Referenzprojekte betrachtet werden, welche nicht – wie es bei Seminararbeiten die gängige Praxis ist – lediglich von Lehrenden gelesen werden, sondern einem breiten Zielpublikum zugänglich sind. Auf der Seite der Lernenden sind die Vorteile insbesondere mit der Möglichkeit verbunden, durch den kostenfreien Zugang Nutzungsbarrieren abzubauen, mit dem Ziel, die systematische Ausgrenzung von einkommensschwachen Gruppen zu vermeiden. Sozialen Ungleichheiten werden so im digitalen Bildungsbereich entgegengewirkt. Durch die globale Nutzung und dem kostenfreien Zugang bieten sich Vorteile nicht nur für sozial schwache Schichten in entwickelten Ländern, sondern auch für Lernende aus Entwicklungsländern.

Als notwendigen nächsten Schritt sehen wir unter anderem die Erstellung einer Übersicht, um die Gesamtsituation der Erstellung und Erweiterung von Kursangeboten in Forschungsprojekten, Universitäten, Schulen besser im Kontext eines Geschäftsmodells zu verstehen und zu analysieren. In dem hier besprochenen Kontext wird die Kurserstellung als Teil des Forschungs- und Bildungsangebots gesehen und ist somit direkt an die bestehenden Informations- und Wertströme gebunden und davon zeitlich und ressourcenmäßig abhängig. Daher ist eine konkrete Darstellung der relevanten Akteur*innen sowie der Informations- und Wertströme notwendig.

Barrieren ergeben sich primär aus dem Bereich des Datenschutzes, diese sind für kommerzielle sowie für nichtkommerzielle Online-Angebote gleich. Bei der Entwicklung und Nutzung sind die Grundsätze der DSGVO verpflichtend. Des Weiteren ist die Einwilligung der Teilnehmenden zur Nutzung ihrer Verhaltensdaten als Opt-In entscheidend. Hinzu kommt, dass die Lernenden sehr gläsern sind (ähnlich wie es bei der gängigen Praxis bei der Websuche der Fall ist), dies jedoch gemäß unserer Umfrage von der Mehrheit akzeptiert wird.

Bei der Erstellung der Onlinekurse sind zudem Urheberrechte, z. B. für Bildmaterialien, zu beachten. Wird das nicht gewährleistet, darf ein Kurs nicht eingesetzt werden, andernfalls wird dessen Veröffentlichung zum Kostenrisiko. Vor allem Studierende müssen für das Thema des Urheberrechts sensibilisiert werden, bevor sie Kurse entwickeln und verbreiten. Eine juristische Prüfung der Kurse kann notwendig sein. Eng damit verbunden ist die Frage nach der Qualität der Kurse im Allgemeinen – das Bewertungs- und Assessmentsystem muss eindeutig und verpflichtend sein. Zusätzliche Schwierigkeiten bei der Datenauswertung zur weiteren Nutzung ergeben sich dadurch, dass Verhaltensdaten grundsätzlich durch den gezielten Einsatz von Bots manipuliert werden können. Hier müssen bereits zu Beginn Mechanismen entwickelt werden, welche dies verhindern. Die Ergebnisse der Umfrage haben gezeigt, dass „gesponsorte“ Inhalte weitgehend akzeptiert werden. Wenn kommerzielle Anbieter Produktplatzierungen zum Zweck des Marketings verwenden möchten, erfolgt dies in der Regel unter der Beachtung der Optimierung der Reichweite. Demnach würden lediglich Kurs-Themen ausgewählt, welche möglichst viele Personen erreichen – andere Themen erhalten aus der Perspektive des Marketings jedoch kaum Beachtung. Im Kontext des GM$ sollte kritisch berücksichtigt werden, dass die Kursanbieter die ethische Verantwortung dafür tragen, Werbeinhalte und -einsatz zu überprüfen und manipulative Inhalte ausschließen. Zusammenfassend ermöglicht das in diesem Beitrag vorgeschlagene zweiseitige Geschäftsmodell eine weitläufig akzeptierte und nachhaltige Verwertung und Bereitstellung von Onlinekursen, deren Trennung von ökonomischen Interessen ein Angebot mit maximaler Qualität ermöglicht.
